# Association of *Salmonella* virulence factor alleles with intestinal and invasive serovars

**DOI:** 10.1186/s12864-019-5809-8

**Published:** 2019-05-28

**Authors:** Alexey V. Rakov, Emilio Mastriani, Shu-Lin Liu, Dieter M. Schifferli

**Affiliations:** 1 Department of Pathobiology, School of Veterinary Medicine, University of Pennsylvania, Philadelphia, Pennsylvania USA; 2 Systemomics Center, College of Pharmacy, Genomics Research Center, State-Province Key Laboratories of Biomedicine-Pharmaceutics of China, Harbin Medical University, Harbin, China; 3 HMU-UCCSM Centre for Infection and Genomics, Harbin Medical University, Harbin, China; 4 Department of Microbiology, Immunology and Infectious Diseases, University of Calgary, Calgary, Canada; 5 Present Address: Somov Institute of Epidemiology and Microbiology, Vladivostok, Russia

**Keywords:** *Salmonella enterica*, virulence factors, allelic variation, host adaptation, FimH, STs, iNTS

## Abstract

**Background:**

The role of *Salmonella* virulence factor (VF) allelic variation in modulating pathogenesis or host specificity has only been demonstrated in a few cases, mostly through serendipitous findings. Virulence factor (VF) alleles from *Salmonella enterica* subsp. *enterica* genomes were compared to identify potential associations with the host-adapted invasive serovars Typhi, Dublin, Choleraesuis, and Gallinarum, and with the broad host-range intestinal serovars Typhimurium, Enteritidis, and Newport.

**Results:**

Through a bioinformatics analysis of 500 *Salmonella* genomes, we have identified allelic variants of 70 VFs, many of which are associated with either one of the four host-adapted invasive *Salmonella* serovars or one of the three broad host-range intestinal serovars. In addition, associations between specific VF alleles and intra-serovar clusters, sequence types (STs) and/or host-adapted FimH adhesins were identified. Moreover, new allelic VF associations with non-typhoidal *S.* Enteritidis and *S.* Typhimurium (NTS) or invasive NTS (iNTS) were detected.

**Conclusions:**

By analogy to the previously shown association of specific FimH adhesin alleles with optimal binding by host adapted *Salmonella* serovars, lineages or strains, we predict that some of the identified association of other VF alleles with host-adapted serovars, lineages or strains will reflect specific contributions to host adaptation and/or pathogenesis. The identification of these allelic associations will support investigations of the biological impact of VF alleles and better characterize the role of allelic variation in *Salmonella* pathogenesis. Most relevant functional experiments will test the potential causal contribution of the detected FimH-associated VF variants in host adapted virulence.

**Electronic supplementary material:**

The online version of this article (10.1186/s12864-019-5809-8) contains supplementary material, which is available to authorized users.

## Background

In recent years, it has been shown that adaptation of pathogen to certain niches is accompanied by genome degradation [1]. One of the models used for this observation is *Salmonella enterica*, a versatile Gram-negative *Enterobacteriaceae*, that includes over 2,500 different serovars and accounts for a vast number of human and animal infections [2, 3]. *S. enterica* strains causing human disease are divided into human-restricted typhoidal serovars (Typhi and Paratyphi) and nontyphoidal (NTS) serovars (e.g. Enteritidis, Typhimurium, Newport and most others *S. enterica* serovars). The latter serovars are designated generalists, since they also infect a broad-range of mammals and birds, causing gastroenteritis and/or persistent infections with potential for long-term shedding. Animals have also more invasive serovars capable of extra-intestinal spread responsible for septicemia, such as *S.* Gallinarum, which is restricted to poultry and many wild birds [4], and *S.* Dublin and *S.* Choleraesuis, that are mostly adapted to cattle and pigs, respectively, with rarer human or other mammalian infections. Some *Salmonella* serovars can cause invasive NTS infections (iNTS) by septicemic spread [5], and are often not associated with diarrhea. In the last decade, such strains were isolated mostly from Africa, frequently in malaria or HIV-infected patients. Genomic studies have shown that they are mainly caused by specific lineages of *S.* Typhimurium [6] and *S.* Enteritidis [7].

From an evolutionary point of view, the most parsimonious explanation for the host-restricted serovar is that they emerged from an ancestor with a broad host range [8]. The *Salmonella* adaptation process to a reduced number of hosts and a more efficient escape from the microbial competitive intestinal milieu by invading deeper tissues resulted in the loss of genes by gradual pseudogenization and subsequent deletions. Nuccio et al. demonstrated that 469 genes of the central anaerobic metabolism network underwent some degree of degradation in extraintestinal *Salmonella* serovars [9]. During the re-annotation of 15 *S. enterica* genomes they found that 471 intact genes had been misidentified as pseudogenes. Despite progress in pseudogene annotation, the precise mechanisms associated with host adaptation remain mostly unexplained. One infrequently addressed caveat with pseudogenes is that some of them still express full proteins due to frameshifting or t-RNA-mediated suppression over premature stop codon, particularly relevant possibilities when only low levels of gene product is required for full function [10, 11].

In addition to pseudogenization and gene loss, host adaptation in *Salmonella* has also been influenced by gene gains via horizontal genetic transfer, such as the acquisition of virulence plasmids and pathogenicity islands that encode genes used for adherence, host cell invasion, induction of innate immune and/or pathophysiological responses [8, 12, 13]. More subtle effects can result from point mutations that modify DNA structure and recognition sites for regulatory binding proteins or cause amino acid substitutions in open reading frames that might contribute to host adaptation of *S. enterica* serovars [14]. Overall, adaptation to human or animal species is a complex phenotype [15] and the relative importance of the participation of gene loss, gain or modification for a preferential intestinal or systemic pathotype remains understudied.

Early investigations using multi locus enzyme electrophoresis and multilocus sequence typing (MLST) already revealed that in contrast to generalists, host-adapted *Salmonella* serovar showed reduced allelic variation in their house-keeping genes [16]. Partial core genome analysis of several generalist and host-adapted *Salmonella* serovars was consistent with the separation of clear lineages distinguishing generalists and specialists [17]. Core or whole genome analyses have been very useful for phylogeny and epidemiological typing [18–20], but their potential to identify critical allelic variants participating in host preference and virulence remains obscured by an excess of mutations that may not be adaptive or relate to other phenotypes, such as survival in different non-host environments. Relevant *Salmonella* properties linked to intestinal immunopathology, septicemic capacities and/or adaptation to certain host species are more likely enriched among allelic variants of virulence genes such as surface or exported proteins that interact with host cells and molecules [14]. Notably, we previously showed that a distinctive genomic property of *S*. Typhimurium’s association with diverse hosts related at least partially to allelic variants of surface or exported proteins, as established with some fimbrial adhesins [21, 22]. Allele-specific adhesive properties confirmed the biological significance of the detected associations. For example, the FimH allele most frequently present on bovine *S.* Typhimurium strains mediated best bacterial binding to bovine intestinal epithelial cells, whereas bacteria expressing a FimH allele associate most frequently with human isolates adhered preferentially to human intestinal epithelial cells. Comparable associations were determined for the host-adapted binding properties of the FimH alleles from other intestinal and invasive serovars, such as Typhi and Choleraesuis. Further studies showed that even though strain-specific combination of alleles for different adhesins of the broad host range serovar Newport mediated preferential binding to one host species, they kept at least one allele mediating binding to cells from another host. These findings suggested that allelic variation in multiple adhesins of a generalist serovar can contribute to bacterial adaptation to certain preferential hosts without losing the capacity to maintain a broad host range [22].

Since *Salmonella* evolution to increased levels of host-adaptation may accompany more aggressive and invasive pathotypes, we hypothesized that not only adhesins, but also other proteins involved in virulence might have allelic variants that are associated with either intestinal or septicemic *Salmonella* serovars or strains. To examine this issue, we collected the sequences of 70 virulence genes from 500 *Salmonella enterica* subsp. *enterica* strains. We deduced the protein sequences of the genes and compared the alleles. To evaluate the potential involvement of allelic variation in the more aggressive and invasive *S.* Typhimurium and Enteritidis iNTS strains, we analyzed them separately. In support of our hypothesis, we found that not only specific degraded/absent virulence gene, but also certain allelic virulence protein sequences were linked to the intestinal or invasive lifestyle of the various *Salmonella* serovars or lineages. These findings narrow down the list of genes and allelic variants that may relate to host adapted pathologies worth studying for host-determined effects. The confirmation of previously described virulence levels associated with specific allelic or absent virulence factors [21, 23–26] supports the potential biological relevance of the new host-virulence factor allele associations detected in this study.

## Results

### Collection of virulence factors from intestinal and systemic *Salmonella* serovars

For this study, 70 known VFs from 500 *Salmonella* genomes were collected and their protein sequences were deduced to analyze and compare their alleles. Selection of the VFs (Additional file 1: Table S1) was based on their presence in most of the serovars studied and their capacity to interact directly with host molecules such as known adhesins and effector proteins exported by the two *Salmonella* type 3 secretion systems (T3SS) [27–29]. We included in the list outer membrane proteins and LPS modifying proteins that are regulated by the PhoPQ two component system which modulates the surface of intracellular *Salmonella* [30, 31]. The analysis included proteins from four host-adapted or restricted invasive serovars and three typical broad host range intestinal serovars (Additional file 2: Table S2).

### Host-related VF gene degradation

**Fig. 1:**
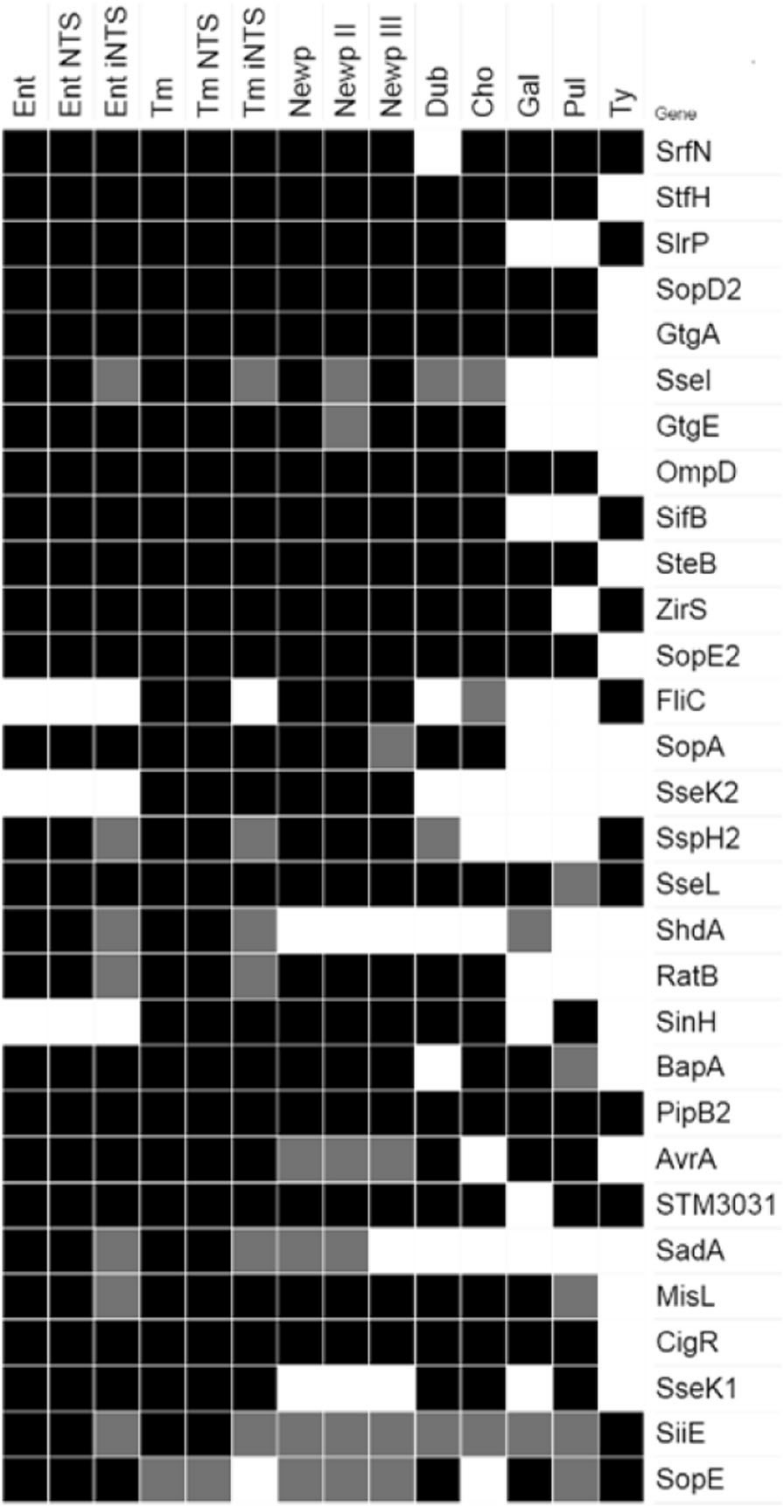
Virulence factor predicted to be missing due to disrupted or absent genes among the genomes of 7 *Salmonella* serovars and some of their lineages. Serovars and lineages are indicated on the top and and VFs on the right. The heatmap shows genes present in most (> 80 %, in black), some (≤ 80%, in grey), or no genomes (white) due to deletion or pseudogenization

**Fig. 2:**
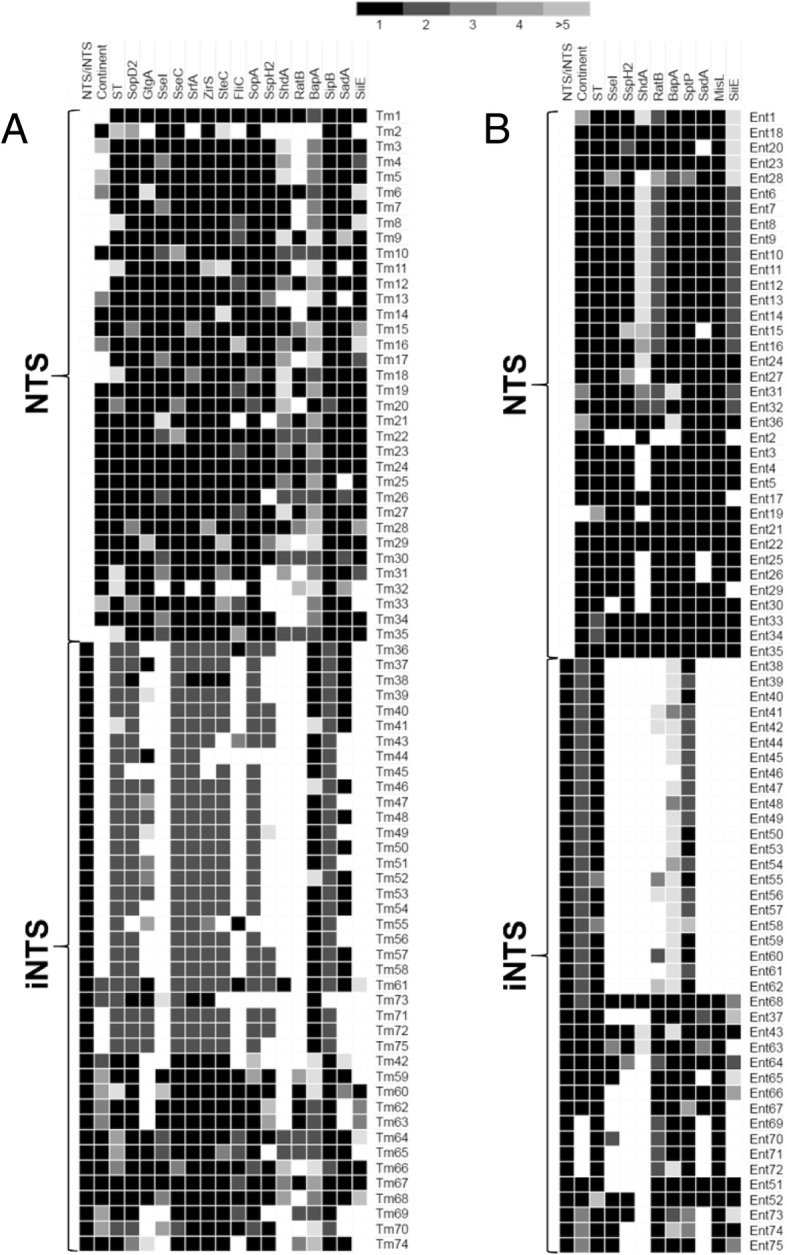
Distribution of the virulence factor alleles most frequently degraded among iNTS and non-iNTS. **a** *S.* Typhimurium and (**b**) *S.* Enteritidis genomes. VFs are shown on the top and strains on the right side. The heatmap shows the numbers of different alleles for each gene product for each strain represented in black (one allele), different gray gradations (2 to ≥ 5 alleles), or white (absent gene product)

The most host-adapted invasive and pseudogenized *S.* Typhi had also the most disrupted or absent VF genes from a collection of 70 selected virulence genes (27.1%) (Additional file 3: Table S3 and Fig. 1). The VF genes of this human restricted serovar underwent even a greater selective pressure than the corresponding genome itself (5-10%)[9, 32]. Similarly, the predicted reduction in VFs for the other three invasive *Salmonella* serovars (Gallinarum, Choleraesuis and Dublin) was significantly higher than that for the three intestinal *Salmonella* serovars (9.5% versus 2.0%; p < 0.0001, two-tailed Fisher’s exact test). Thus, in addition to the inactivation of genes for unnecessary metabolic pathways used for intestinal colonization [9, 32], many VFs of intestinal *Salmonella* generalists are apparently superfluous and possibly deleterious for invasive-septicemic infections. In addition, the more host-restricted serovars Typhi and Gallinarum had less than half as many predicted virulence genes as the host-adapted serovars Choleraesuis and Dublin, including the SPI-2 effector proteins SseI/SrfH, GtgE, SopA, SseK2, and the outer membrane proteins RatB and SadA (Additional file 3: Table S3). Concurring findings were obtained with increased tendency for VF gene degradation of iNTS versus non-invasive NTS from serovars Typhimurium and Enteritidis (Fig. 2a and b), in agreement with earlier whole genome studies [7, 33]. By including only one sub-Saharan representative of each serovar to minimize same strain/lineage over-representation [6, 7] statistically significant differences in gene degradation between iNTS and non-invasive NTS was found for SseI, SspH2, ShdA, SadA and SiiE (p < 0.02) in *S.* Enteritidis, and SseI, ShdA and SiiE (p < 0.01) in *S.* Typhimurium (Fig. 2a and b, Additional file 3: Table S3). *S.* Typhimurium iNTS of the ST313 lineage showed also increased degradation for SspH2 and SadA as compared with non-invasive NTS in general (p < 0.0001). All sub-Saharan *S.* Enteritidis iNTS strains lacked MisL, unlike all the other iNTS strains (p < 0.0001) and all non-invasive NTS strains, suggesting that they may form a separate lineage. Although *Salmonella* pseudogenization has generally been recognized as a successful evolutionary adaptation to increased virulence for fewer hosts as well as reduced or lost virulence for generalists [9, 32], as shown for SseI in specific infection models [23, 34–36], the biological relevance of most pseudogenized or missing genes remains to be demonstrated.

### Allelic variation among the 70 VFs

**Fig. 3:**
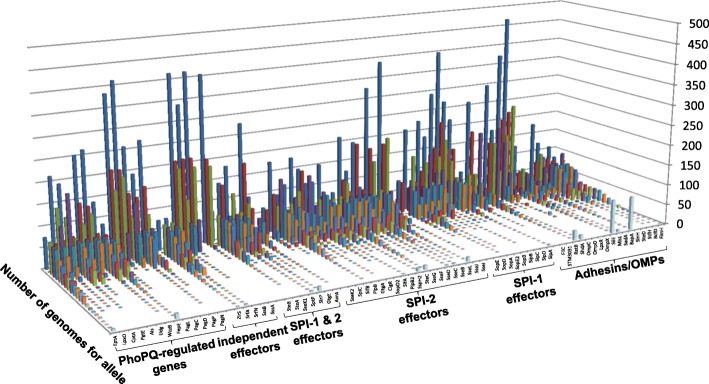
Allelic frequencies for 70 studied virulence factors among 500 *Salmonella*. VFs are organized in six groups as labeled on the bottom of the graph (PhoPQ-regulated, “independent”, SPI1 and/or SPI2 effectors, adhesins/outer membrane proteins). Each VF has several alleles whose number of strains per allele are each represented by column heights, as labeled on the right axis. The most frequent to the least frequent allele (from allele 1 to > 20) are shown from the back to the front of the graph, each in a different color (as best visualized with the first VF, EptA, on the left)

DNA sequence changes were evaluated in 70 genes encoding VFs and the corresponding deduced protein sequences were used for all the comparative analyses of this study. Protein alignments identified allelic protein variants that were assigned incremental numbers (Additional file 4: Table S4). Comparisons were based on protein and not gene sequences to detect potentially functional associations with different pathotypes of the *Salmonella* serovars and lineages. The number of amino acid substitutions in the deduced protein sequences was compared between each allele and the dominant allele of the corresponding 70 proteins. We calculated Hamming distances (number of amino acid changes between the most common allele and other alleles for each of the studied protein) and found the PhoPQ-regulated proteins were the least diverse (Additional file 5: Figure S1). As can be seen from the figure, the most variable VFs were StfH, BapA, SiiE, RatB, FliC, SteC, SlrP and ZirS. The most diverse proteins, as determined by the Simpson’s Diversity index, were StfH, SlrP, SteA, ZirS, SteC, FliC, SspH2, SseL, RatB, BapA and SiiE (Additional file 6: Table S5). The number of alleles varied from 4 to 98, with a mean value of 9.4 ± 2.1 per protein (confidence level 95%; median value of 16). Most proteins with the highest numbers of alleles were high molecular weight membrane proteins such as SiiE (595 kDa), RatB (206 kDa) and BapA (386 kDa). The ratio of VF alleles to the total residues per VF varied from 0.8% (SadA) to 15% (SssA), with a mean of 5.5% (Additional file 6: Table S5). On average, 11.9% of the residue positions varied for all VF, with a median value of 7.4%. Nearly a third (32.2%) of all the VF were represented by one dominant allele, defined as including at least 50% of the strains (Fig. 3). Notably, adhesins FimH, BcfD, StfH, SteD, SinH, BapA, SadA, MisL and SiiE were more variable than other VFs, as determined by genetic diversity *h* [37] with a mean of 35.6 for the adhesin alleles and 16.7 for the other VF alleles (p = 0.08, Mann–Whitney U test; Additional file 7: Table S6). A few proteins had two major alleles, such as the fimbrial adhesins BcfD, StfH and FimH, the large membrane proteins ShdA, RatB, BapA, CigR and SiiE, the secreted proteins SlrP, SseI, PipB, SifA, SpiC, SseC, SteA, SrfA, SteC, SopA, SseL, SopE and ZirS, and the outer membrane protein OmpD (Fig. 3). Some proteins (21%) had a most frequent allele for less than 20% of the strains, including the high molecular weight membrane-associated protein ShdA, SiiE, BapA and SadA. In summary, *Salmonella* VFs demonstrate a significant level of allelic variation.

### VF alleles in intestinal and invasive *Salmonella* serovars

Even though dominant alleles of most VFs were shared almost equally between the intestinal and invasive serovars of *Salmonella* (Additional file 8: Figure S2), some dominant protein alleles were only present in the former (StfH, SseI, SifB, SopA, SseK2, ShdA, BapA, SadA, SiiE and SopE) or latter serovars (FimH, SlrP, SteA, SrfA, ZirS, SteC, FliC, SspH2, SseL, RatB and MgtB). Of the 70 proteins studied, 58 (83%) had alleles that were shared between two or more serovars. In contrast, 16 proteins had serovar-restricted alleles, the adhesin FimH, the flagellin FliC, the membrane-associated proteins ShdA, RatB, SadA, SiiE and BapA, the PhoPQ-regulated protein EptA, and the secreted proteins SteA, SrfA, SteC, SopA, PipB2, SseK1, SseK2 and ZirS. The alleles of the phage-encoded SopE were shared only between intestinal serovars. Allelic variation of each protein within one serovar was evaluated by calculating *h* values (Additional file 7: Table S6). Comparisons of monoallelic proteins (*h* = 0) suggested that the intestinal serovars were twice as diverse as the invasive serovars (Additional file 7: Table S6, mean number of monoallelic VFs = 30.3 versus 13.2, respectively; p = 0.01, Mann–Whitney U test). Moreover, dominant protein alleles from either intestinal or invasive serovars with *h* values below 0.1 confirmed a corresponding trend (mean number of VFs with *h* < 0.1 = 43.2 versus 32.7 alleles; p = 0.25 Mann–Whitney U test). Even though host-restricted *S.* Typhi had over three alleles for 17 VFs (Additional file 7: Table S6), it was the least diverse serovar with the largest number of monoallelic proteins (65.7%), followed by the other invasive serovars (38.7%) and the intestinal serovars (18.9%). Taken together, these results support a *Salmonella* evolutionary model whereby the invasive serovars are younger than the intestinal serovars, with the accumulation of allelic variation being time dependent. In addition, purifying selection linked to host adaptation most likely participated in restricting allelic variation.

Among all the VFs, SteC and FimH had the highest Simpson’s diversity indices with values close to one (> 0.9, Additional file 6: Table S5). Linkage disequilibrium (LD) was evaluated by determining indices of associations (I_AS_) and mean gene product diversity (H) for each serovar, Newport lineages and Gallinarum biovars (Additional file 7: Table S6). LDs correlated with gene product diversities. Diversity increased from serovar Typhi to serovars Newport, consistent with the previously described polyphyletic nature of the latter serovar [38, 39]. Interestingly, biovar Pullorum showed greater VF allelic variability than biovar Gallinarum; this might be related to a broader host range, particularly in wild birds, of the former biovar [40].

To evaluate whether alleles of the 70 VFs can be used to identify subdivisions among all *Salmonella* studied irrespective of serovars, a range of assumed population groups (K = 2-13) was investigated, and K = 10 was found to be the best estimate to discriminate serovars and lineages with the Structure v. 2.3.4 program [41–43] (Additional files 9 and 10: Figures S3 and S4). In addition to recognizing the individual serovars, the program distinguished the two known Newport lineages and Gallinarum biovars at this K value, with a separate cluster for strains in lineage III of *S.* Newport. A minimum spanning tree obtained with goeBURST located the intestinal serovars in the central nodes and the invasive serovars on tip nodes (Additional file 11: Figure S5), and thus best illustrated how the invasive serovars represent dead-ends in pathogen evolution as they relate to host adaption. In summary, VF variants were found associated with unique sets of flagellar and O-antigens by separating the seven serovars and to further divide two serovars in their biovars or lineages, in agreement with their distinct evolutionary host adaptations.

### Hierarchical clusters of *Salmonella* VF alleles

**Fig. 4.**
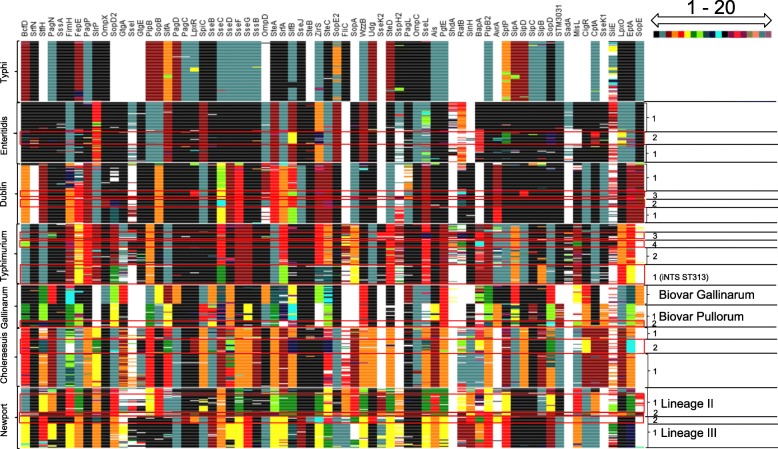
Heatmap showing hierarchical clustering of 500 *Salmonella* strains with corresponding alleles across the 70 virulence factors studied. The seven serovars each with 52-75 strains are shown on the left of the heatmap, VFs on the top, arrayed in the order genes are located on the chromosome. Clade numbers, biovars (*S.* Gallinarum) and lineages (*S.* Newport) are shown on the left. Colors indicate different alleles, with black being the dominant allele for each VF among all 500 *Salmonella*; blue-green, the 2^nd^ most frequent allele, etc. up to the 20^th^ (and more) most frequent allele in purple, as shown on the top left (1-20); missing VFs are shown in white

Hierarchical clustering analyses were performed to determine the distribution of VF alleles among the different serovars. The colored heat map showing each VF’s 20 most dominant alleles irrespective of serovars not only confirmed that in contrast to all other serovars Typhi has more deleted or pseudogenized VF genes, but also showed that Typhi has unique alleles for 66% of its VFs (Fig. 4). Significant variation was only detected in the repetitive bacterial immunoglobulin domains of the giant Ca-binding inner membrane adhesin SiiE [44]. The heat map also illustrated the highly clonal property of *S.* Typhi, in agreement with earlier whole genome studies [45].

*S.* Enteritidis was divided in two clusters of different alleles for the seemingly unrelated proteins SifB, ZirS, BapA, SptP and SopD (Fig. 4). The smaller cluster 2 consisted of half of the Malawian iNTS strains with a signature allele for the T3SS effectors SptP, SifB, SopD and ZirS, and the PhoPQ-regulators LpxO and CptA (p < 0.05). The other Malawian iNTS and the non-African iNTS isolates belonged to cluster 1, suggesting that separate and/or additional routes of genomic evolution can lead to an invasive phenotype with the likely participation of a weakened host immune system related to concomitant infections or malnutrion [46].

For *S.* Dublin, hierarchical clustering detected minor cluster 3 of human isolates from Uruguay that was characterized by unique VF alleles of SipD and LpxR (Fig. 4). MLST did not distinguish these strains in earlier studies [47, 48].

Two major *S.* Typhimurium groups could be distinguished by hierarchical clustering with one group subdivided in three smaller clusters (Fig. 4). The majority of *S.* Typhimurium iNTS lineage consisted of the African iNTS isolates (ST313 and ST2080), represented by cluster 1. All the ST313 strains had different alleles for SopD2, GtgA, SseC, SopA and SipB (p < 0.03). The other few iNTS were dispersed in cluster 2, 3 and 4.

Hierarchical clustering clearly separated *S.* Gallinarum biovar Gallinarum from biovar Pullorum with separate alleles for 23 VFs (StfH, FimH, OmpX, SopD2, GtgA, PipB, SopB, SifA, PagD, SseB, SseC, OmpD, SteA, SrfA, SifB, ZirS, SteC, PagL, PipB2, AvrA, SipA, SopD and CigR), as well as the presence or absence of STM3031and SseK1 (Fig. 4). Biovar Pullorum was further divided in two clusters that had different alleles for 11 VFs (BcfD, StfH, SpiC, SseB, SseC, SseG, SifB, Ais, PipB2, SipC and SopD), whereas SrfA was absent in one cluster. Cluster 2 of this biovar shared several alleles with biovar Gallinarum (BcfD, SpiC, SseG and Ais).

*S.* Choleraesuis was divided in two hierarchical clusters distinguished by FimH, StfH, SifA, LpxR, SifB, IagB, IacP, SipB, SpaR, SopD and MgtB (Fig. 4). The two clusters appeared to not overlap with the annotation for the two major biovars of Choleraesuis, senso stricto and Kunzendorf (Additional file 2: Table S2). However, since a recent study discovered that there were many errors for the biovar annotations and that all *fimH*^*-*^ strains were biovar senso stricto [49], our data suggest that all biovar senso stricto strains belong to cluster 2.

For *S.* Newport, hierarchical clustering of VF alleles detected two major groups (Fig. 4) that corresponded to the known lineages II and III of this serovar [38]. Lineage II included an additional discrete cluster of Asian isolates recognizable by a novel StfH allele and separate alleles for SrfN, SteA, RatB, SinH, AvrA, MisL, CigR and SopE. Lineage III had a separate cluster of strains that were characterized by specific alleles for FimH, SlrP, PagC, LpxR, SteB, ZirS, SteC, Udg, SinH, BapA, SadA and PhoN, and by the presence of AvrA. As expected, none of the strains studied related to the rare Europe-associated *S.* Newport lineage I [38, 50].

Taken together these results showed that hierarchical clustering of *Salmonella* virulence protein sequences complements whole genomes studies by proposing the identification of new minor strain clusters of potential pathobiological relevance.

### VF alleles associations with STs, adhesin alleles and hosts

Several *Salmonella* serovars and some intra-serovar strain lineages or sequence types are known to demonstrate different levels of host specificities [14, 51, 52]. We previously reported that *Salmonella* strains and serovars with particular adhesin alleles that are more frequently isolated from certain hosts than others typically mediated better bacterial binding to enterocytes from these hosts [21, 22]. Here we suggest parallel host-associations for VF alleles as determined by their genomic ties with alleles of the FimH adhesin. Since an excessive number of the randomly selected strains from this study lacked the required metadata, specifically the source of isolation (Additional file 2: Table S2), we relied on our previously described adhesin allele-host associations to propose potential roles of VF alleles in host-specific virulence.

Two serovars, Typhi and Enteritidis, had no significant FimH allelic variation and thus no potentially possible host associations with VF alleles. *S.* Typhi had only one FimH allele for all 75 genomes evaluated, and similarly, *S.* Enteritidis had one dominant FimH allele (92% of the strains) with only four new minor alleles mostly in Malawian strains that were not associated with STs or other VF alleles (Additional file 2: Table S2 and Additional file 12: Figure S6). This was consistent with the host restriction or preference of both serovars, Typhi as a human pathogen and Enteritidis as an effective bird colonizer and poultry-derived food product contaminant responsible for human infections.

**Fig. 5:**
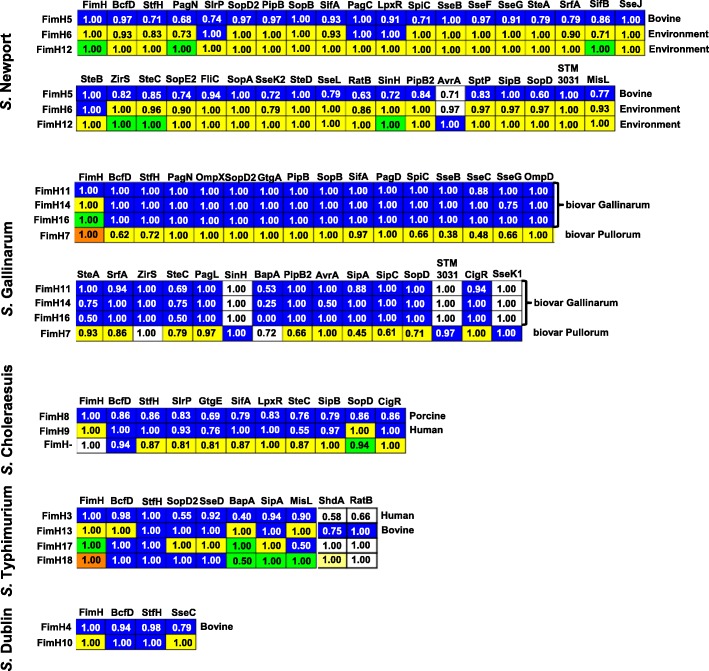
Degree of association of FimH alleles with other virulence factor alleles for five *Salmonella* serovars. VF alleles are indicated on the top of each heatmap while on the left the major FimH alleles (Additional file 7: Table S6) to which they are associated in *S.* Newport, *S.* Gallinarum, *S.* Choleraesuis, *S.* Typhimurium, or *S.* Dublin. Different alleles are shown in different colors (allele 1 to 4, blue, yellow, green and brown, respectively). Missing VFs are shown in white. Numbers indicate the prevalence of the most dominant allele for the corresponding serovar/lineage

*S.* Dublin isolates were mainly ST10 strains (93.3% of all isolates) (Additional file 2: Table S2). A newly observed second FimH allele (30.7% of 75 strains; Additional file 12: Figure S6) associated with an allele of SseC (Fig. 5) with no detectable host preference.

Most *S.* Typhimurium were either non-invasive NTS ST19 or iNTS ST313 strains (Additional file 2: Table S2) with mainly the human-associated FimH alleles [21]. A few ST19 strains formed cluster 4 (Additional file 12: Figure S6 and Additional file 2: Table S2) and had the FimH allele previously found to be preferentially associated with bovines and to bind best to bovine intestinal epithelial cells [21]. These strains also had unique alleles for the surface-exposed proteins BcfD, BapA, ShdA, RatB and MisL (Fig. 5), suggesting that like FimH, the five other proteins might participate in some level of adaptation to the bovine host. In addition to the previously described five FimH alleles one new minor allele was discovered (Fig. 5, Additional file 2: Table S2).

In addition to different sequence types (Additional file 2: Table S2), *S.* Gallinarum biovars Gallinarum and Pullorum had different alleles for 44% of the VFs. Strains of biovar Gallinarum had either one of three FimH alleles (Additional file 2: Table S2), even though most of their other VFs were represented by only one allele. In contrast, biovar Pullorum had a fourth FimH allele with mostly different VF alleles than biovar Gallinarum (Fig. 5, and Additional file 12: Figure S6), which likely relates to the different pathogenesis of these bird-restricted biovars [53]. Although all discovered FimH, StfH, and BcfD adhesin alleles were not shared across serovars, the BcfD allele 1 was an exception by being shared between both biovars of *S.* Gallinarum and *S.* Enteritidis, potentially participating in the concurring affinity of these serovars for avian hosts [54].

The majority of *S.* Choleraesuis of known origin were porcine isolates that included mainly ST145 and ST66 strains (Additional file 2: Table S2 and Additional file 13: Figure S7). All *S.* Choleraesuis strains of cluster 1 (Additional file 12: Figure S6) had either one of the two previously reported FimH alleles, the porcine-associated FimH104 (here Fim8) or the human-associated FimH105 (here FimH9) [21]. Even though there were very few confirmed human isolates in the current study to corroborate these associations [21], the two FimH alleles were each present with a different SopD allele (Fig. 5). Cluster 2 strains (21.6%) consisted of both of human and porcine isolates lacking *fimH* and typically associated with specific alleles, as shown in Fig. 4 (Additional file 2: Table S2).

MLST delineated *S.* Newport lineages II and III, in agreement with the previously described host [38, 55, 56] and allelic adhesin associations [22]. The two lineages were also differentiated by distinct alleles for many VFs (Additional files 11 and 12: Figures. S5 and S6, Additional file 2: Table S2). Consistent with previous findings, lineage II was mostly populated by strains with the three adhesin alleles A/A/A1 for FimH/BcfD/StfH whereas the lineage III strains had mainly alleles B/B/B1 for these adhesins [22]. Lineage II formed the largest cluster that mainly consisted of ST45 strains which were typically US isolates. A few ST31 and ST46 strain (together with its single locus variants ST157, ST2364) were from Asia (Additional file 12: Figure S6 and Additional file 2: Table S2), with unique alleles for ST31 (MisL, SseL, PipB2, CigR, SteA) and all these Asian strains (StfH, SinH, SiiE, RatB, AvrA, LpxO). Lineage III had two separate clusters of VFs mainly populated by either ST5 (21 strains in cluster 1) or ST118 strains (16 strains in cluster 2). The small cluster 2 of lineage III included strains with distinct set of alleles and the consistent presence of AvrA (Additional files 11 and 12: Figures S5 and S6, Additional file 2: Table S2).

Finally, to determine whether VF generally show more allelic variation than other gene products, we evaluated the protein sequences of 40 other gene products chosen to be only indirectly associated to virulence as a stringent comparative group (Additional file 14: Table S7). Most serovars, lineages or biovars showed significant greater allelic variations among the 70 VFs than their corresponding control group, in support of their potential role in host adaptation (Additional file 15: Figure S8). In addition, the VF alleles that formed different strain clusters and serovars that concomitantly share reported host-associated allelic FimH might represent further protein sequence adaptations of VFs to certain hosts and pathotypes, as suggested for adhesin alleles [14, 21, 22].

## Discussion

Having previously confirmed the biological relevance of detected associations of *Salmonella* adhesin alleles with host species [21, 22, 27], we extended our studies by interrogating the extent of protein sequence variabilities of 70 VFs in 500 genomes from 7 *Salmonella* serovars. Strains from both intestinal generalists and invasive-septicemic host-adapted serovars were evaluated. Distinct new allelic protein variants and gene losses/pseudogenes were observed to be associated not just with specific serovars or a few STs, as previously described [9, 47, 57], but more broadly with many unique STs and with strain lineages or clusters within one serovar. For example, by screening the genes encoding VFs, we found that *shdA* and *siiE* were pseudogenized in all isolates belong to *S*. Typhimurium ST313 lineage, in addition to confirming the previously described inactivation of the genes for SseI, RatB, and SspH2 for ST313 strains [6]. Whether *shdA* and/or *siiE* pseudogenization plays a role in host adaptation or systemic infections remains to be determined. Most significantly, we found that in addition to *sadA*, *sseI* and *siiE* were also pseudogenized in the sub-Saharan African S. Enteritidis iNTS strains, and we confirmed the reported pseudogenes for SspH2, ShdA, and RatB in these strains [7]. More generally, gene degradation was found to be significantly higher for VFs than previously reported with whole genome studies on host restricted and adapted serovars. In addition to pseudogenization, a decreasing VF allelic variability from intestinal broad host range to invasive/septicemic host-adapted serovars highlighted the involvement of virulence factor alleles in evolutionary adaptation to fewer hosts with the concomitant change in virulence and disease severity.

The role of pseudogenization in changing *Salmonella* into a systemic pathogen has been well described. For example, pseudogenization of *sopD2*, *sopA* and s*opE2* in *S.* Typhi increased pathogen invasiveness [24, 26]. Similarly, whereas SseI supported a generalist’s persistence in its host by inhibiting dendritic cell migration [35], its absence participated in the spreading of iNTS strain 313 in the host [23]. However, the latter example might be *Salmonella* strain and infection model-dependent, since SseI was previously shown to participate in invasiveness [34–36].

Although *shdA* which encodes a Peyer’s patch and cecum colonization factor was shown to be pseudogenized in the typical host adapted serovars [3, 48], a pseudogenized *shdA* in *S.* Typhi was shown to be functional [25]. Apparently, shorter proteins and/or bypassed stop codons by translational frameshifts with potential recoding can result in functional proteins [10]. Thus, even though the *shdA* gene was pseudogenized in most iNTS of serovars Enteritidis (92%) and Typhimurium (87%), and in all but one *S.* Newport strains, it remains possible that a functional ShdA protein is expressed in theses strains. Alternatively, loss of ShdA-mediated colonization in some strains might be compensated by the activity of one or more other bacterial factors [22].

Each VF showed an extensive range of allelic variants, most being shared between 2 or more separate serovars and only 17% being serovar-restricted. Analysis of the VF allelic variants by three methods separated the serovars from one another, including the Newport lineages and Gallinarum biotypes, suggesting a co-adaptative evolution between the *Salmonella* VF alleles and its O- and flagellar antigens that define its serovars. Results from the goeBURST program visualized the intestinal generalists as nodes on a central branch with branches radiating separately out of it for each host-adapted invasive serovar, in support of separate adaptation histories for their VF alleles. Hierarchical cluster analysis further separated VF alleles into different clusters. Interestingly, both serovar Typhimurium and Enteritidis iNTS formed separate clusters that included either only iNTS strains, or both iNTS and non-invasive NTS. Hence, iNTS comprise both successful outbreak strains such as ST313, as well as epidemically less efficient iNTS whose invasive classification is likely based on clinical symptoms of sepsis, as related to a weakened immune system of individual patients. Serovar Choleraesuis was clearly separated in two clusters by two main sets of VFs alleles, in contrast to serovar Dublin which had one main set of VFs, with a few strains forming 2 minor clusters. Allelic profiles of these host-adapted *Salmonella* might relate to host-determined variables such a pig and cattle breeds [58], and differential geographic distribution of livestock breeds. As expected, the two biovars of serovar Gallinarum formed separate clusters, albeit biovar Pulllorum showed more diversity with an additional sub-cluster. However, each biotype had relatively few alleles for each VF, in agreement with their adaptation to the restricted avian host, and their rare isolation from wild birds [59]. *S.* Newport has the most sets of diverse VF alleles, with lineages II and III each having two separate clusters. One clade and a subclade of lineage II were mainly occupied by Asian isolates from humans or edible non-mammalian vertebrates, suggesting parallel adaptation to different geographical niches with different dietary sources [60].

In addition to the described presence of specific VF alleles in ST313 of iNTS, several major STs of serovar Typhimurium, Gallinarum, Choleraesuis and Newport showed associations with sets of VF alleles. However, the separate evolution of intra-serovar lineages and within some STs was better revealed by the numerous sets of allelic VF variants that are potentially involved in successful adaptations to unique or multiple distinct niches. A physical association between the alleles of three fimbrial adhesin genes in 262 *S.* Newport isolates previously showed two major sets of allelic variants for most strains, one representative of bovine and porcine isolates, and the other of environmental and human isolates [22]. Each strain had at least two allelic adhesins binding significantly better than the other alleles to an intestinal epithelial cell line from the original host(s), leaving one adhesin allele binding best to corresponding cells from another host. Thus, allelic variation in multiple adhesins of S. Newport was suggested to contribute to bacterial adaptation to certain preferential hosts while still maintaining a large host range [22]. Based on these results and the established genomic link between FimH and other VF alleles for each of the 500 strains of this study, we inferred that some of the VF alleles were also host-associated, and possibly host-adapted. Accordingly, sets of VF alleles, each with an associated host-adapted FimH were found in serovar Typhimurium, Choleraesuis, Gallinarum and Newport.

Although this study hints at the possibility that some of the described serovar, lineage, biotype and clonal associations for VF alleles might play a role in host specificity and pathogenesis, the biological relevance of the detected associations need to be tested for a definite proof, as addressed previously for a few adhesins [21, 22, 61]. It is also expected that a significant number of amino acid substitutions in various alleles are essentially silent with no functional effect on host adaption. Other substitutions might have indirect effects. For example, different SptP alleles between *S.* Typhi and Typhimurium affect export efficiency of this SPI-1 effector protein [62]. Some of the variable amino acids of the SipD tip protein of *S.* Typhi and Typhimurium might not represent adaption to different pathogen-host interfaces, but provide recalibrated contacts with variable PrgI needle subunit proteins for structural assembly and function of the type 3 secretion system [63]. Lastly, amino acid replacements might also inactivate the function of the protein and represent a first steps in pseudogene formation.

To optimize the significance of our comparative analyses and minimize biases, 500 genomes were collected randomly, choosing similar numbers for each serovar, biotype or Newport lineages, and for Typhimurium and Enteritidis iNTS and non-invasive NTS, based genome data availability. Taxonomically misidentified genomes were rejected and several serovar Gallinarum biovars were classified by their genomic signatures [64]. Unfortunately, many *Salmonella* genomes lacked metadata on host origins for the corresponding isolates, with the possibility that some strains were epidemiological clones. However, when clones were suspected as for the African ST313 isolates, only one representative strain was used for comparisons. A main strength of this study was the inclusion of a relatively large number of *Salmonella* VFs and genomes from several intestinal and septicemic serovars. This allowed us not only to confirm a few previous observations on allelic VF variants, but also to bring attention to VFs whose sequences that associate with specific metadata might play a role in the different pathogenic properties of the various serovars and strain lineages.

## Conclusions

In conclusion, the comparative analysis of the allelic variants of 70 VFs from three intestinal/broad range *Salmonella* serovars and four invasive/septicemic serovars is providing new information on amino acid substitutions that might modulate the function of *Salmonella* proteins known to play a role in host specificity and pathogenesis. These findings should help to better target wet lab and animal model experiments aimed at identifying allelic variants responsible for causal effects.

## Methods

### Bacterial genomes

A total of 500 *Salmonella enterica* subsp. *enterica* genomes were used for the study: 75 *S.* Enteritidis, 75 *S.* Typhimurium, 74 *S.* Newport, 75 *S.* Dublin, 74 *S.* Choleraesuis, 75 *S.* Typhi, and 52 *S.* Gallinarum biovars Gallinarum and Pullorum strains (Additional file 2: Table S2). Genomic data with corresponding metadata were downloaded from NCBI RefSeq database. Raw sequencing data with metadata were collected from NCBI SRA or EBI ENA repositories and were assembled using SPAdes genome assembler implemented through Galaxy/GVL server [65]. Sampling was performed randomly with higher assembly level being collected first. With the exception of *S*. Gallinarum that had only 52 available genomes 75 genomes were collected from each serovar. Incorrectly serotyped strains were determined for one *S.* Newport and one *S.* Choleraesuis genome, as determined by their sequences with SISTR [19], which found that 5% of all *Salmonella enterica* genomes or short reads deposited in publicly available databases were misidentified. The MLST v. 1.8 server was used for the detection of sequence types (ST) [66]. ST and metadata for some of the genomes were obtained from EnteroBase [67].

We collected and translated 70 genes for known and putative *Salmonella* VFs [29], including adhesins, SPI-1 and SPI-2 effectors and PhoPQ-regulated proteins (Additional file 1: Table S1). A control group of 40 genes, encoding proteins indirectly related to virulence were listed in the same table.

### VF allele’s determination

Most protein sequences were identified with TBLASTN (e-value cut-off of 0.001, minimum query coverage >95%), and additional proteins were detected with MegaBLAST, using *S.* Typhimurium LT2 (Genbank # NC_003197) nucleotide sequence information (e-value cut-off of 0.01, minimum query coverage of >95% and identity >95%). Translation from DNA to protein sequences was performed in MEGA7 [68]. Multiple sequence alignment was performed in BioEdit v. 7.2.6.1 (Ibis Biosciences, an Abbott Co.). The SignalP v. 4.1 server was used for the prediction and location of signal peptide cleavage sites [69]. Variable amino acid residues were extracted from protein alignments using FaBox v. 1.41 online tool [70].

Alleles were defined as protein sequences without signal peptide, which have at least one amino acid change (nonsynonymous single nucleotide polymorphism; nsSNP) or indel enumerated incrementally in order of their frequency. A script was made to number protein allelic variants and calculate Hamming distances (number of amino acid substitutions between sequences) for each allelic protein of the 500 genomes to determine dominant allele(s) [71].

### Association analysis and statistics

The standardized index of association, I_AS_, was used as a measure of multilocus linkage disequilibrium (LD) [37]. The null hypothesis of linkage equilibrium, I_AS_ = 0, was tested with 10,000 Monte Carlo simulations using the LIAN v. 3.7 program [37]. Allelic diversity at each locus (h) was also estimated using LIAN. The mean of the two groups (“intestinal” vs. “invasive”) of allelic frequencies was compared using a Mann–Whitney U test performed with Prism v. 6.0 (GraphPad Software Inc.). P-values that compared allele numbers from iNTS vs. non-iNTS isolates were computed by 2x2 contingency table with the two-tailed Fisher’s exact test in QuickCalcs (GraphPad Software Inc.). P-values for the genetic diversity comparisons between the 70 virulence factors and 40 virulence-associated proteins within each serovar, biotype or lineage studied were done with an unpaired t-test using Prism v. 6.0.

### Population and phylogenetic analysis

Global optimal eBURST (goeBURST) algorithm implemented by PHYLOViZ Online (N locus variant = 25) was used for phylogenetic inference and data visualization of minimum spanning tree [72]. To evaluate population structure, we used Structure v. 2.3.4 [43]; four independent runs were performed for each value of the number of populations K ranging from 2 to 13. Each run consisted of 100,000 Markov Chain Monte Carlo iterations (the first 50,000 being discarded as burn-in iterations). We used the Evanno △K method [41] representing the highest median likelihood values with the online CLUMPAK server [42]. The Comparing Partitions online tool was used for calculating Simpson’s diversity indexes [73] with confidence interval (CI)[74]. CINA is non-approximated CI from the original Simpson study [75]. Other metrics were calculated from sequence information for each VF (number of aminoacid for the mature VF, number of alleles divided by the total number of amino acids, number of variable amino acids, number of variable amino acids divided by the total number of amino acids).

### Visualization softwares

All heatmap images were produced by using the complete linkage method with Euclidian distance metric for hierarchical clustering of the Morpheus server (Broad Institute, MIT, https://software.broadinstitute.org/morpheus ). Population structure results were visualized with the CLUMPAK server [42]. The Venn diagram was made by eulerAPE v. 3 [76].

## Additional files

Additional file 1: **Table S1.** List of virulence factors and “control” proteins selected for this study. (XLSX 20 kb)

Additional file 2: **Table S2.** List of the 500 *Salmonella* genomes (7 serovars) selected and metadata. (XLSX 58 kb)

Additional file 3: **Table S3.** List of virulence factor genes absent in some *Salmonella* strains or serovars. Genes were either present (+) or absent (-) in all or some strains (+/-). (XLSX 14 kb)

Additional file 4: **Table S4.** List of allele numbers for the 70 virulence factors in the 500 *Salmonella* strains. (XLSX 114 kb)

Additional file 5: **Figure S1.** Allelic diversity for 70 studied virulence factors among 500 *Salmonella* assessed by Hamming distance. The heatmap shows VFs represented as columns and organized in six groups as labeled on the top of the heatmap. Distance corresponds to gradient colors varied from blue (main allele/no amino acid changes) to red (most amino acid changes). Missing VFs are shown in white. (PPT 264 kb)

Additional file 6: **Table S5.** Genetic diversity for 70 virulence factors among all serovars. Simpson’s index with confidence interval (CI), non-approximated confidence interval (CINA), number of amino acids (aa) in the mature protein (without the signal peptide, SP), number of alleles to the number of total amino acids, number of variable amino acids, percent of variable amino acids per total amino acids. (XLSX 17 kb)

Additional file 7: **Table S6.** Genetic diversity for each serovar, lineage or biotype. Mean genetic diversity, and index of association I_AS_, were determined by the LIAN v. 3.7 software. Number of monoallelic VFs, VFs with allelic diversity h < 0.1, total number of monoallelic VFs and VFs with allelic diversity h < 0.1, were calculated with corresponding 95% confidence intervals. Cells were highlighted with a color gradient (white = 0 to darkest blue = 0.8655). (XLSX 15 kb)

Additional file 8: **Figure S2.** Distribution of dominant alleles versus other alleles for 70 studied virulence factors. VFs are represented as horizontal bars and organized in six groups as labeled on the left side of the graph. The numbers of dominant alleles are shown separately for the intestinal serovars in green (*S.* Typhimurium, *S.* Enteritidis and *S.* Newport*)* and the invasive serovars in red (*S.* Typhi, *S.* Dublin, *S.* Gallinarum, *S.* Choleraesuis); all the other alleles are grouped and shown in white. Bars that do not reach 500 (number of *Salmonella* studied) represent missing VFs. (PPT 100 kb)

Additional file 9: **Figure S3.** Best stratified population based on 70 virulence factor sequences from 500 *Salmonella*. The best estimated K value was equal to 10, as calculated by the Evanno △K plot that represents the highest median likelihood values for each K, using the CLUMPAK server. (PPT 64 kb)

Additional file 10: **Figure S4.** Population stratification by 70 virulence factor sequences from 500 *Salmonella*. The range of numbers of assumed populations K was tested from 2 to 13 as shown on the left using the Structure 2.3.4 program. The 70 VF alleles of 500 *Salmonella* were grouped by serovars, and lineages (*S.* Newport) or biotypes (*S.* Gallinarum), as shown on the top, and represented by thin vertical lines. The coloring of each vertical line was proportional to the ancestry of each isolate for each K population. (PPT 202 kb)

Additional file 11: **Figure S5.** Minimum spanning tree based on the alleles of the 70 virulence factors. The minimum spanning tree was built with the goeBURST algorithm, using an N locus variant level equal to 25 to link all nodes with distances equal or above this level. The tree created nine clusters represented as red circle for the generalists (*S.* Enteritidis, *S.* Typhimurium, *S.* Newport lineage II and *S.* Newport lineage III) and as black circles for the septicemic serovars (*S.* Dublin, *S.* Choleraesuis, *S.* Gallinarum biovar Gallinarum, *S.* Gallinarum biovar Pullorum, and *S.* Typhi). Circle sizes correspond to the number of strains for each cluster. (PPT 91 kb)

Additional file 12: **Figure S6.** Reorganized heatmap of the 70 virulence factors and 40 control gene products. This heatmap was based on the function of the virulence factors and using the hierarchical clustering data of the 500 *Salmonella* strains as determined in Fig. 4. In addition to the VFs studied, 40 gene products for virulence-associated proteins were evaluated as a comparative group**.** The 7 serovars each with 52-75 strains are shown on the left of the heatmap. VFs on the top, arrayed in 7 functional groups, starting with FimH, BcfD and StfH in the adhesin group and ending with the comparative group. Clade numbers, biovars (*S.* Gallinarum) and lineages (*S.* Newport) are shown on the left. Colors indicate different alleles, with black being the dominant allele for each VF among all 500 *Salmonella*; blue-green, the 2^nd^ most frequent allele, etc. up to the 20th (and more) most frequent allele in purple, as shown in Fig. 3 (1-20); missing VFs are shown in white. (PPT 1058 kb)

Additional file 13: **Figure S7.** Proportional Venn diagram for STs and their FimH alleles in *S.* Choleraesuis. The diagram displays the number of strains with a specific ST and FimH allele. As an example, for ST145 strains, 23 have a predicted FimH8 allele (red), 16 have a predicted FimH9 allele (blue-purple) and 1 is predicted to lack FimH (yellow), whereas all 13 ST66 strains are predicted to have the FimH9 allele. (PPT 82 kb)

Additional file 14: **Table S7.** Genetic diversity for 40 virulence factors-associated proteins among all serovars. Values as described in Additional file: Table S5. (XLSX 12 kb)

Additional file 15: **Figure S8.** Mean genetic diversity (H) of 70 virulence factors (blue) and 40 virulence-associated proteins (red) for each serovar/lineage/biotype studied. The two groups H values were compared using an unpaired t-test. Statistically significant differences are marked by asterisks. (PPT 115 kb)

## Data Availability

Not Applicable.

## Abbreviations

(i)NTS: (invasive) non-typhoidal *Salmonella*
I_AS_: Index of association
LD: Linkage disequilibrium
MLST: Multilocus sequence typing
*S. enterica*: *Salmonella enterica*
SNP: Single nucleotide polymorphism
ST(s): Sequence type(s)
T3SS: Type 3 secretion system
VF(s): Virulence factor(s)
