# Supplementary material for: Association of Salmonella virulence factor alleles with intestinal and invasive serovars
Source: BMC Genomics. 2019 May 28;20:429. doi: 10.1186/s12864-019-5809-8 (PMC6540521; doi:10.1186/s12864-019-5809-8)
Supplement: Supplementary file 5 — Figure S1. Allelic diversity for 70 studied virulence factors among 500 Salmonella assessed by Hamming distance. The heatmap shows VFs represented as columns and organized in six groups as labeled on the top of the heatmap. Distance corresponds to gradient colors varied from blue (main allele/no amino acid changes) to red (most amino acid changes). Missing VFs are shown in white. (PPT 264 kb) [file 12864_2019_5809_MOESM5_ESM.ppt]

## Slide 1
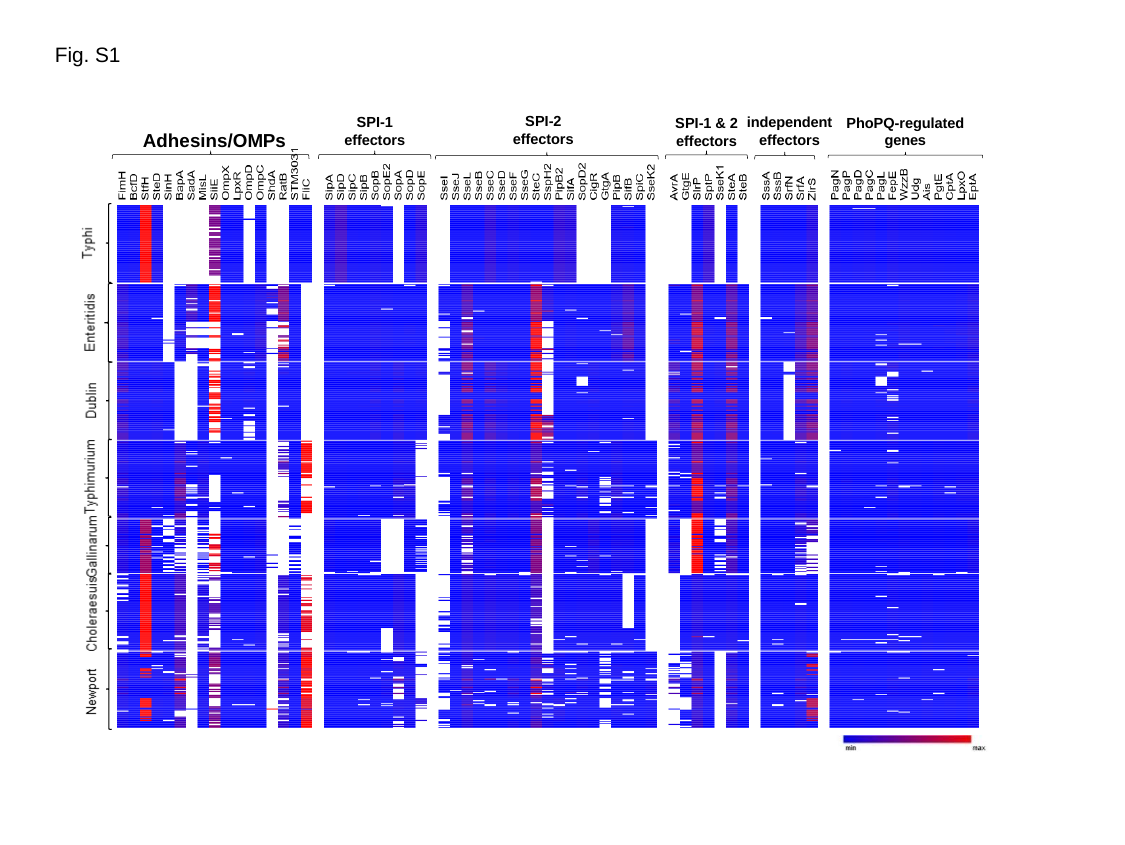

Fig. S1
SPI-2
effectors
SPI-1
effectors
independent
effectors
PhoPQ-regulated
genes
SPI-1 & 2
effectors
Adhesins/OMPs
