# Supplementary figures and images for: Association of Salmonella virulence factor alleles with intestinal and invasive serovars
Source: BMC Genomics. 2019 May 28;20:429. doi: 10.1186/s12864-019-5809-8 (PMC6540521; doi:10.1186/s12864-019-5809-8)

## Slide 1
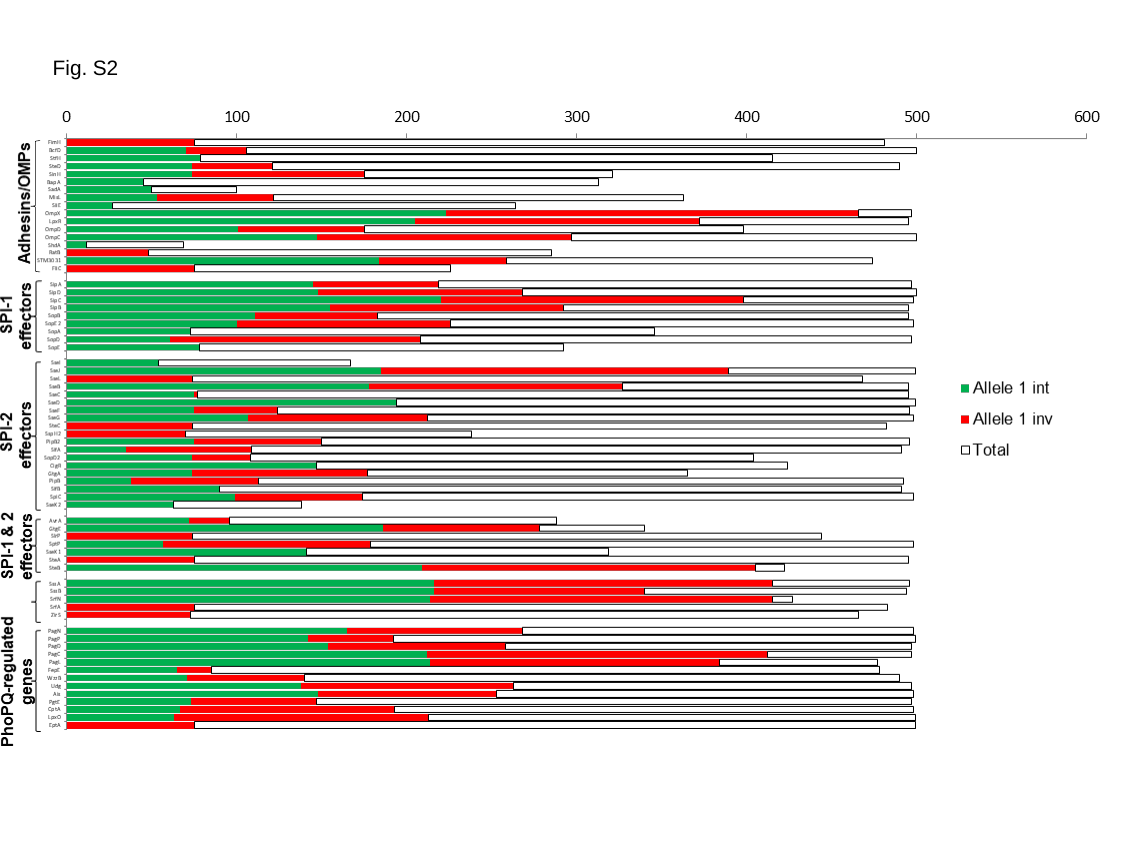

Fig. S2

Supplement: Supplementary file 8 — Figure S2. Distribution of dominant alleles versus other alleles for 70 studied virulence factors. VFs are represented as horizontal bars and organized in six groups as labeled on the left side of the graph. The numbers of dominant alleles are shown separately for the intestinal serovars in green (S. Typhimurium, S. Enteritidis and S. Newport) and the invasive serovars in red (S. Typhi, S. Dublin, S. Gallinarum, S. Choleraesuis); all the other alleles are grouped and shown in white. Bars that do not reach 500 (number of Salmonella studied) represent missing VFs. (PPT 100 kb) [file 12864_2019_5809_MOESM8_ESM.ppt]

## Slide 1
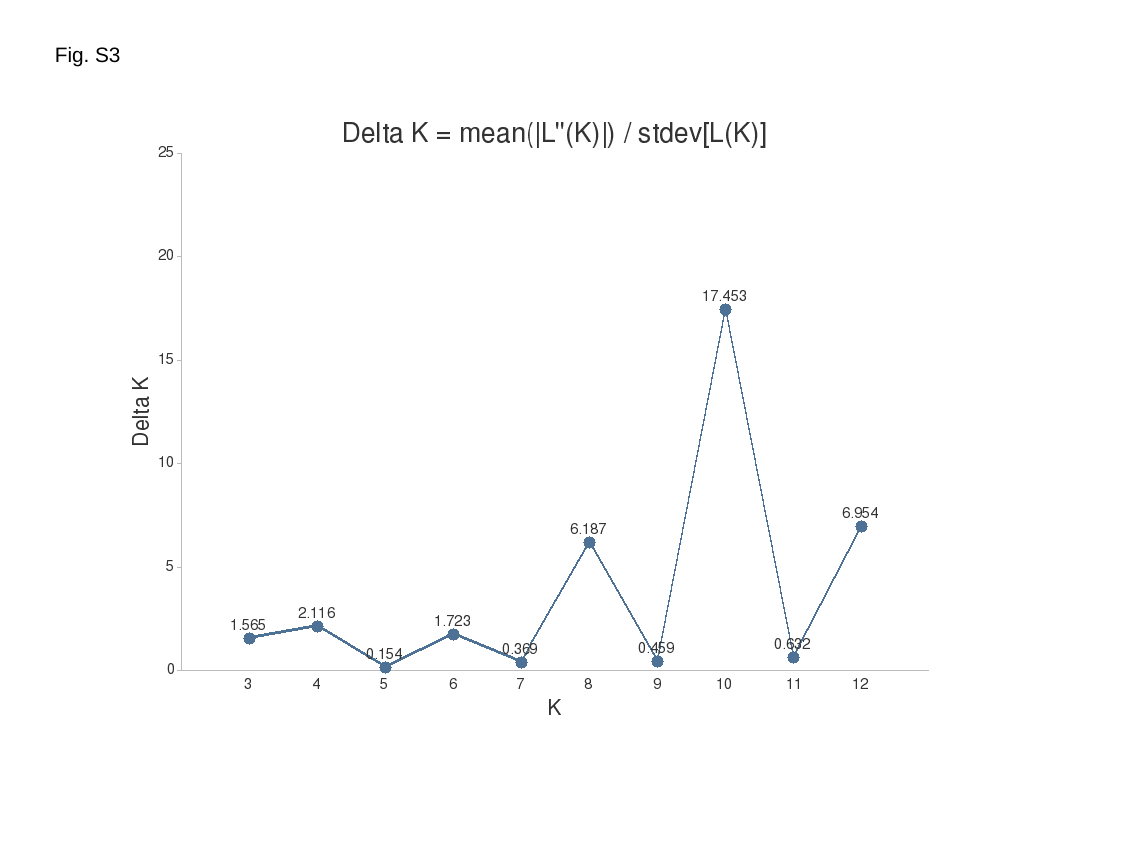

Fig. S3

Supplement: Supplementary file 9 — Figure S3. Best stratified population based on 70 virulence factor sequences from 500 Salmonella. The best estimated K value was equal to 10, as calculated by the Evanno △K plot that represents the highest median likelihood values for each K, using the CLUMPAK server. (PPT 64 kb) [file 12864_2019_5809_MOESM9_ESM.ppt]

## Slide 1
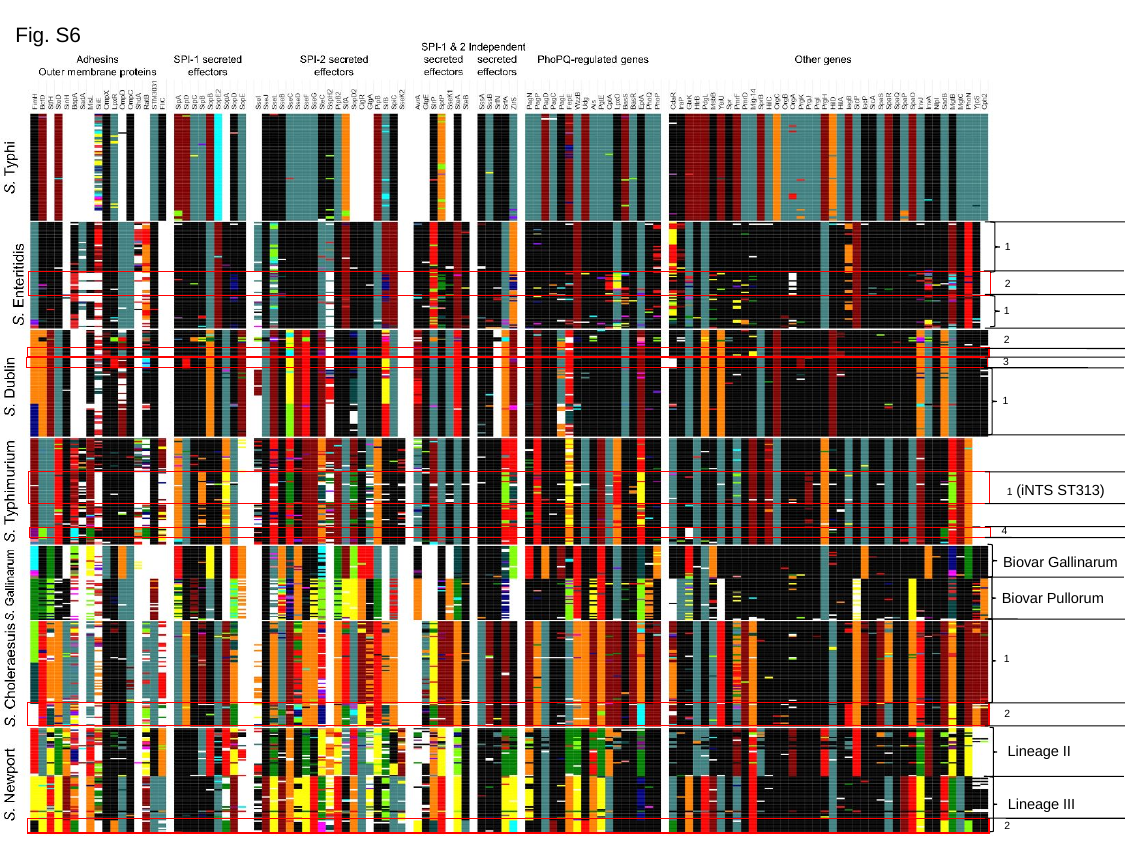

Fig. S6
1
2
1
2
3
1
1 (iNTS ST313)
4
Biovar Gallinarum
Biovar Pullorum
1
2
Lineage II
Lineage III
2

Supplement: Supplementary file 12 — Figure S6. Reorganized heatmap of the 70 virulence factors and 40 control gene products. This heatmap was based on the function of the virulence factors and using the hierarchical clustering data of the 500 Salmonella strains as determined in Fig. 4. In addition to the VFs studied, 40 gene products for virulence-associated proteins were evaluated as a comparative group. The 7 serovars each with 52-75 strains are shown on the left of the heatmap. VFs on the top, arrayed in 7 functional groups, starting with FimH, BcfD and StfH in the adhesin group and ending with the comparative group. Clade numbers, biovars (S. Gallinarum) and lineages (S. Newport) are shown on the left. Colors indicate different alleles, with black being the dominant allele for each VF among all 500 Salmonella; blue-green, the 2nd most frequent allele, etc. up to the 20th (and more) most frequent allele in purple, as shown in Fig. 3 (1-20); missing VFs are shown in white. (PPT 1058 kb) [file 12864_2019_5809_MOESM12_ESM.ppt]

## Slide 1
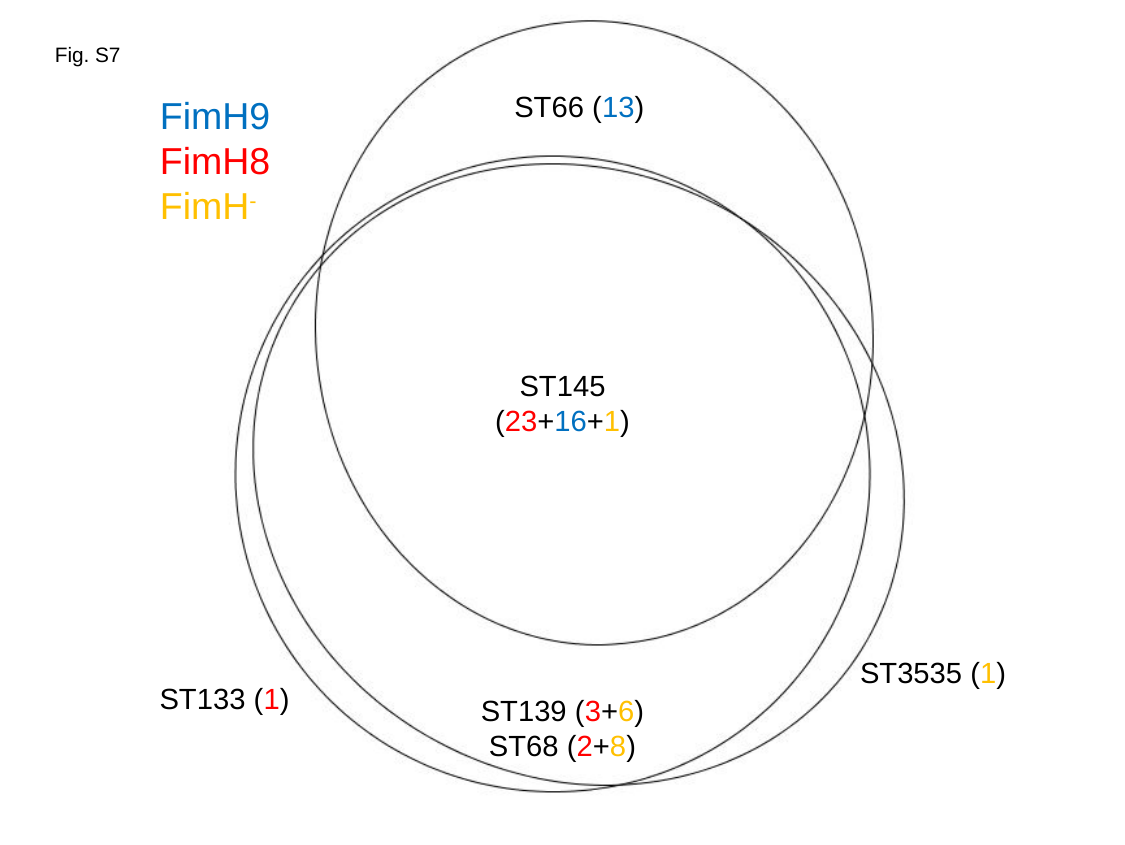

Fig. S7
ST66 (13)
FimH9
FimH8
FimH-
ST145
(23+16+1)
ST3535 (1)
ST133 (1)
ST139 (3+6)
ST68 (2+8)

Supplement: Supplementary file 13 — Figure S7. Proportional Venn diagram for STs and their FimH alleles in S. Choleraesuis. The diagram displays the number of strains with a specific ST and FimH allele. As an example, for ST145 strains, 23 have a predicted FimH8 allele (red), 16 have a predicted FimH9 allele (blue-purple) and 1 is predicted to lack FimH (yellow), whereas all 13 ST66 strains are predicted to have the FimH9 allele. (PPT 82 kb) [file 12864_2019_5809_MOESM13_ESM.ppt]

## Slide 1
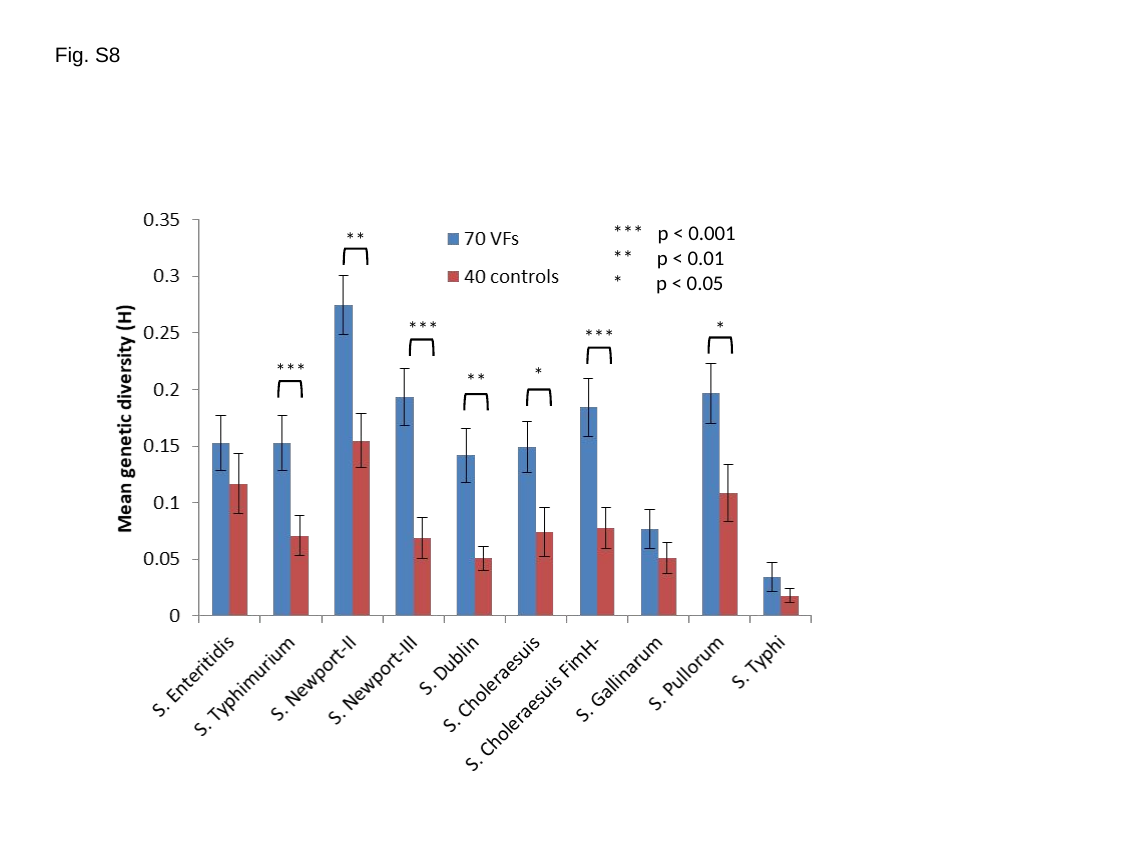

Fig. S8
*** p < 0.001
** p < 0.01
* p < 0.05
**
***
*
***
***
*
**

Supplement: Supplementary file 15 — Figure S8. Mean genetic diversity (H) of 70 virulence factors (blue) and 40 virulence-associated proteins (red) for each serovar/lineage/biotype studied. The two groups H values were compared using an unpaired t-test. Statistically significant differences are marked by asterisks. (PPT 115 kb) [file 12864_2019_5809_MOESM15_ESM.ppt]
