# Supplementary material for: Association of Salmonella virulence factor alleles with intestinal and invasive serovars
Source: BMC Genomics. 2019 May 28;20:429. doi: 10.1186/s12864-019-5809-8 (PMC6540521; doi:10.1186/s12864-019-5809-8)
Supplement: Supplementary file 10 — Figure S4. Population stratification by 70 virulence factor sequences from 500 Salmonella. The range of numbers of assumed populations K was tested from 2 to 13 as shown on the left using the Structure 2.3.4 program. The 70 VF alleles of 500 Salmonella were grouped by serovars, and lineages (S. Newport) or biotypes (S. Gallinarum), as shown on the top, and represented by thin vertical lines. The coloring of each vertical line was proportional to the ancestry of each isolate for each K population. (PPT 202 kb) [file 12864_2019_5809_MOESM10_ESM.ppt]

## Slide 1
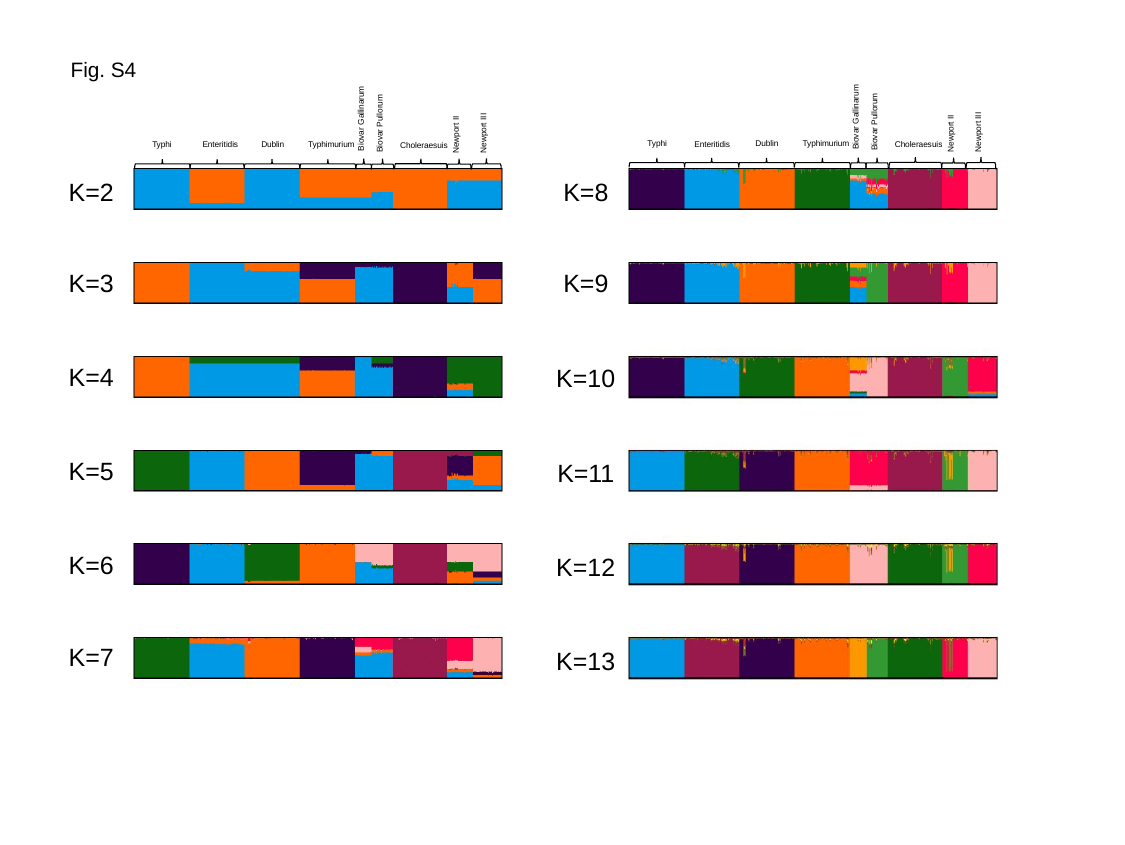

Fig. S4
Biovar Gallinarum
Biovar Gallinarum
Biovar Pullorum
Biovar Pullorum
Newport III
Newport III
Newport II
Newport II
Typhi
Dublin
Typhimurium
Choleraesuis
Enteritidis
Typhi
Enteritidis
Dublin
Typhimurium
Choleraesuis
K=2
K=8
K=3
K=9
K=4
K=10
K=5
K=11
K=6
K=12
K=7
K=13
