# Supplementary material for: Association of Salmonella virulence factor alleles with intestinal and invasive serovars
Source: BMC Genomics. 2019 May 28;20:429. doi: 10.1186/s12864-019-5809-8 (PMC6540521; doi:10.1186/s12864-019-5809-8)
Supplement: Supplementary file 11 — Figure S5. Minimum spanning tree based on the alleles of the 70 virulence factors. The minimum spanning tree was built with the goeBURST algorithm, using an N locus variant level equal to 25 to link all nodes with distances equal or above this level. The tree created nine clusters represented as red circle for the generalists (S. Enteritidis, S. Typhimurium, S. Newport lineage II and S. Newport lineage III) and as black circles for the septicemic serovars (S. Dublin, S. Choleraesuis, S. Gallinarum biovar Gallinarum, S. Gallinarum biovar Pullorum, and S. Typhi). Circle sizes correspond to the number of strains for each cluster. (PPT 91 kb) [file 12864_2019_5809_MOESM11_ESM.ppt]

## Slide 1
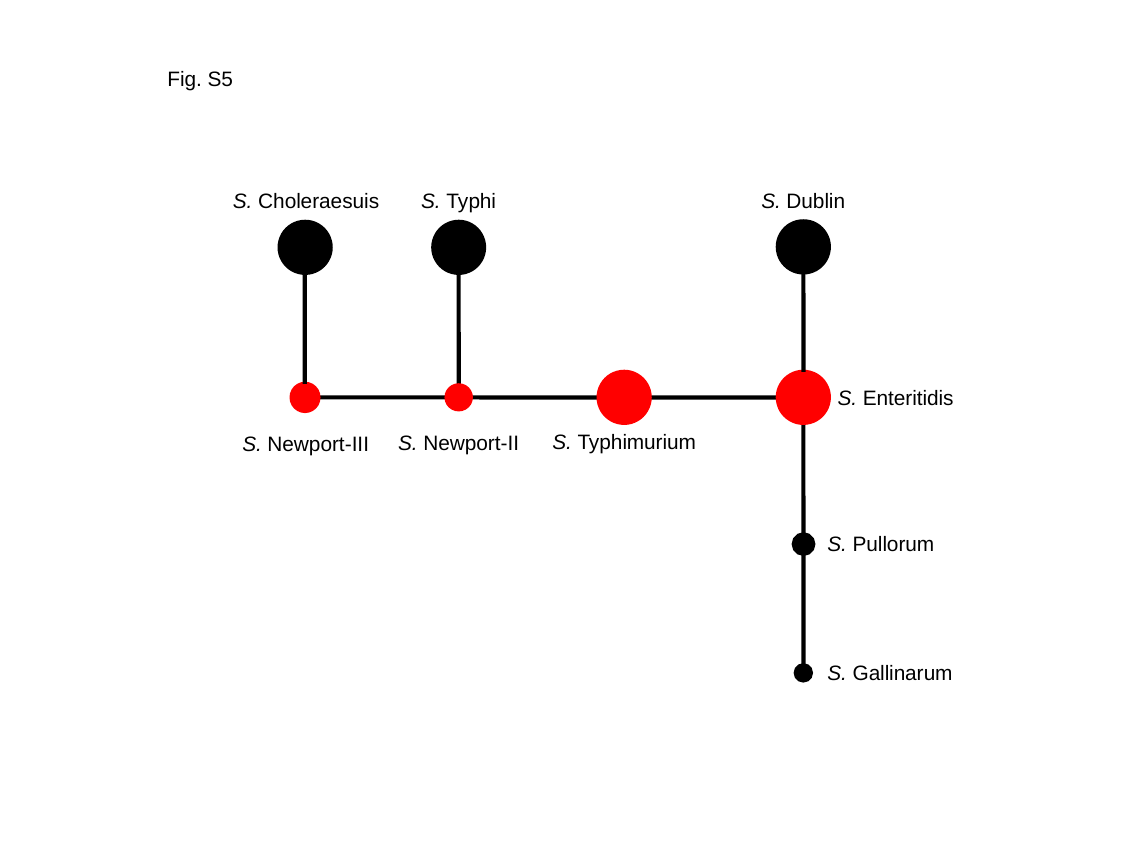

Fig. S5
S. Choleraesuis
S. Typhi
S. Dublin
S. Enteritidis
S. Typhimurium
S. Newport-II
S. Newport-III
S. Pullorum
S. Gallinarum
